# Enhanced Photoacoustic Response by Synergistic Ag–Melanin Interplay at the Core of Ternary Biocompatible Hybrid Silica-Based Nanoparticles

**DOI:** 10.1021/acsami.3c13523

**Published:** 2023-09-29

**Authors:** Brigida Silvestri, Paolo Armanetti, Giulio Pota, Giuseppe Vitiello, Alessandro Pezzella, Luca Menichetti, Vincenzo Giannini, Giuseppina Luciani

**Affiliations:** † Department of Civil, Architectural and Environmental Engineering, University of Naples Federico II, Via Claudio 21, 80125 Fuorigrotta, Naples, Italy; ‡ Institute of Clinical Physiology, National Research Council, Via Giuseppe Moruzzi 1, 56124 Pisa, Italy; § Department of Chemical, Materials and Production Engineering, University of Naples “Federico II”, p.le V. Tecchio 80, 80125 Naples, Italy; ∥ CSGI, Consorzio interuniversitario per lo sviluppo dei Sistemi a Grande Interfase, Sesto Fiorentino, via della Lastruccia 3, 50019 Firenze, Italy; ⊥ National Interuniversity Consortium of Materials Science and Technology (INSTM), Via G. Giusti 9, 50121 Florence, Italy; # Institute for Polymers, Composites and Biomaterials (IPCB), CNR, Via Campi Flegrei 34, I-80078 Pozzuoli (NA), Italy; ∇ Department of Physics Ettore Pancini, University of Naples “Federico II” Via Cintia 4, I-80126 Naples, Italy; ○ Instituto de Estructura de la Materia (IEM), Consejo Superior de Investigaciones Científicas (CSIC), Serrano 121, Madrid 28006, Spain; ◆ Technology Innovation Institute, Building B04C, P.O. Box, Abu Dhabi 9639, United Arab Emirates

**Keywords:** Photoacoustic Imaging, melanin, silver nanoparticles, plasmonic resonance, hybrid nanostructures

## Abstract

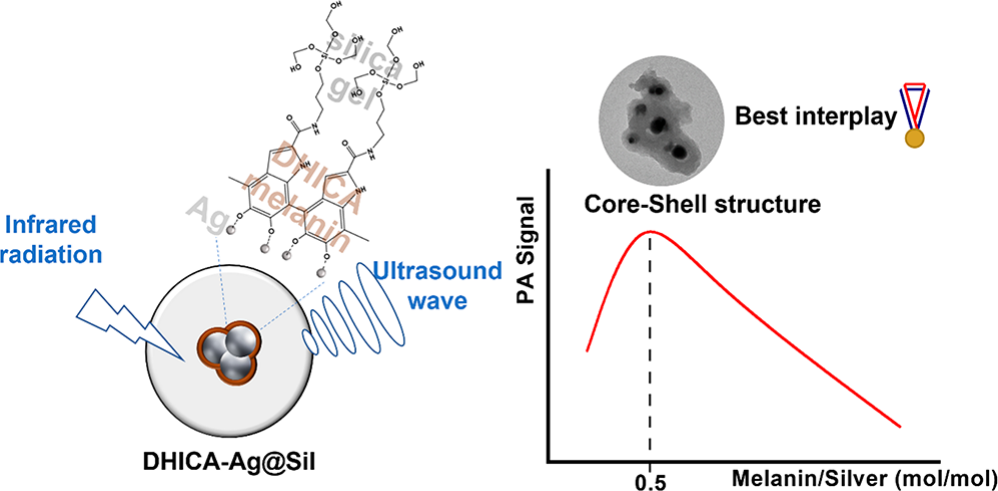

Photoacoustics (PA) is gaining increasing credit among biomolecular imaging methodologies by virtue of its poor invasiveness, deep penetration, high spatial resolution, and excellent endogenous contrast, without the use of any ionizing radiation. Recently, we disclosed the excellent PA response of a self-structured biocompatible nanoprobe, consisting of ternary hybrid nanoparticles with a silver core and a melanin component embedded into a silica matrix. Although preliminary evidence suggested a crucial role of the Ag sonophore and the melanin-containing nanoenvironment, whether and in what manner the PA response is controlled and affected by the self-structured hybrid nanosystems remained unclear. Because of their potential as multifunctional platforms for biomedical applications, a detailed investigation of the metal–polymer–matrix interplay underlying the PA response was undertaken to understand the physical and chemical factors determining the enhanced response and to optimize the architecture, composition, and performance of the nanoparticles for efficient imaging applications. Herein, we provide the evidence for a strong synergistic interaction between eumelanin and Ag which suggests an important role in the in situ-generated metal–organic interface. In particular, we show that a strict ratio between melanin and silver precursors and an accurate choice of metal nanoparticle dimension and the kind of metal are essential for achieving strong enhancements of the PA response. Systematic variation of the metal/melanin component is thus shown to offer the means of tuning the stability and intensity of the photoacoustic response for various biomedical and theranostic applications.

## 1 Introduction

Bioimaging technologies offer tremendous opportunities for noninvasive or minimally invasive monitoring of functional and pathological conditions, thus enabling early diagnosis and more specific therapy which are fundamental for a successful treatment.^1^ Among imaging techniques, photoacoustic imaging is emerging as a powerful hybrid modality, able to combine the advantages of both optical and acoustic signals, thus enabling higher temporal and spatial resolution with good sensitivity and deeper penetration depth.^1^ Furthermore, it can be easily integrated with a photothermal treatment for theragnostic purposes. In this context, the development of functional nanomaterials as contrast agents would provide significant improvement of image quality and diagnosis accuracy,^2^ greatly expanding the functions of the modality.^3^ In view of the multiple features required, contrast agents are usually multicomponent systems obtained by the combination of different materials and exhibit a well-defined structure. Gold nanoparticles have been widely exploited as PA contrast agents.^4−6^ Despite being cheaper than gold, silver nanoparticles have been poorly investigated as PA contrast agents because of their lower performance and poor stability in saline solution, which often requires surface passivation or modification to avoid fast dissolution.^3^ Molecular combination of silver nanostructures with an optically active agent is bound to be an effective strategy to enhance PA properties;^7^ even though it has been poorly investigated mainly following an empirical approach, very few studies focused on unveiling the role of each component and the molecular interface on the PA signal.^7,8^

Among bioactive moieties, scientific attention has been recently focused on melanins, one of the main biological polymers present in most living species, from animals to plants, usually associated just with the color of pigmentation. Actually, melanins play significant roles in several physiological processes, showing photoprotection properties against solar radiation, radical scavenging, camouflage mechanisms in nature, and also body thermoregulation, providing correct homeostasis.^9^ Thanks to their physical and chemical properties, including the broad-light absorption spectrum, metal ion chelating, electrical conductivity, and paramagnetic behavior, melanins have bioinspired a new class of multifunctional materials.^10−13^ Notably, the unique optoelectronic properties of synthetic melanins have recently extended their use to optical theranostics, including fluorescence imaging, photoacoustic imaging (PAI), and photothermal therapy.^14−21^ These features currently provide a fundamental line of research, leading to a high signal-to-noise-ratio (SNR) for deep tissue imaging in the different anatomical districts. Tuning the melanin supramolecular structure through metal ion interaction is a key parameter to drive melanin biological properties and functions. In this prospect, templated polymerization of melanogenic precursors, e.g., 5,6-dihydroxyindole-2-carboxylic acid (DHICA) in the presence of a nanostructured ceramic phase, has recently been disclosed as a purposeful entry into innovative melanin-based hybrid materials with enhanced biofunctional properties and performances.^22−26^ Among inorganic components, SiO_2_ stands as an outstanding platform for biomedical applications in view of its versatility, biocompatibility, and bioactivity as well as the ability to improve chromophore stability and signal amplitude.^27^ The inherent potential of this approach was corroborated by the design and synthesis of ternary silver (core)–melanin/silica (shell) nanoparticles via Ag^+^ reduction during synthesis with as low as 7 wt % eumelanin, exhibiting a stable and enhanced photoacoustic response relative to pure eumelanin or silver nanoparticles. Under pulsed and continuous laser stimulation, these hybrid nanostructures proved to be NIR sensitive, with a relative maximum peak around 700 nm. Moreover, under continuous laser illumination, a dynamic increase of the photoacoustic (PA) signal correlated with sample heating was recorded. Assessment of the PA stability of nanoarticles suspensions indicated a stable and homogeneous distribution pattern of the response with a constant linear intensity of the signal along with the transaxial view.^27^

Preliminary data suggested synergism between the silver nanoparticles and eumelanin in a confined core–hybrid shell architecture as a key structure parameter for enhanced PA efficiency far exceeding that of bare eumelanin–silica particles, enabling contrast detection with definitely low eumelanin concentrations. The particular stability to aggregation of these architectures, coupled with (a) the possibility to fine control morphology and stability to aggregation, thus ensuring cellular uptake, (b) the efficient conjugation of silica to optical active molecules, providing multifunctional capabilities and allowing for specific recognition and selective localization, and (c) the complete biocompatibility ensured by silica, prompted us to undertake further investigations aimed at disentangling and optimizing the silver–eumelanin–silica interaction toward the realization of advanced hybrid nanoparticles for multimodal imaging.

These properties are particularly intriguing because the proximity of plasmonic surfaces of small plasmonic metal nanoparticles, such as those available in the ternary system, can strongly influence the photophysics of melanin. This type of ultrasmall plasmonic nanoparticle also promotes the generation of highly localized regions of intense local field enhancement (hot spots) caused by localized plasmon resonance^28^ which may serve as a trigger for developing new applications in the biomedical field.

Herein, we elucidate the critical physical and chemical determinants of the synergistic interaction between the eumelanin component and Ag in the silica phase. Notably, a theoretical model based on MIE scattering has been developed and allowed us to unveil the contribution of melanin and metal components and their close interaction in determining the overall localized plasmon resonance leading to an amplified photoacoustic effect in these hybrid nanostructures. Furthermore, the model results have been integrated with a detailed physicochemical investigation to support a formation mechanism of nanostructures, able to account for the key role played by each component in the in situ self-assembly process, leading to a core–shell structure, which is endowed with enhanced PA performance.

Systematic variation of the metal/melanin component is also investigated for scaling this effect and assessing the stability and intensity of the PA readout, thus adding tunability to the existing set of favorable features of these nanostructures and paving the way to engineer bioinspired and biocompatible nanomaterials for multimodal imaging and theranostics.

## 2 Materials and Methods

### 2.1 Materials

N-(3-(Dimethylamino)propyl)-N′-ethyl-carbodiimide hydrochloride (EDC, protein seq grade), N-hydroxysuccinimide (NHS, 98%), 3-(aminopropyl)triethoxysilane (APTS), tetra ethyl orthosilicate metal basis (TEOS, 99.999%), ammonium hydroxide solution (NH_4_OH, ACS reagent 28%–30%), ethanol (absolute, ≥99.8%), rhodamine B isothiocyanate (RBITC), silver nitrate (99.9999%), and propidium iodide (PI) were purchased from Sigma-Aldrich (St Louis, MO, USA) and used as received. 5,6-Dihydroxyindole-2-carboxylic acid (DHICA) monomer was prepared as described elsewhere.^29^ Phosphate-buffered saline (PBS) was purchased from Gibco (Grand Island, NY, USA). The live cell labeling kit CytoPainter was purchased from ABCAM (Cambridge, UK). The CellTiter-Glo luminescent cell viability assay was purchased from Promega (Madison, WI, USA).

### 2.2 Synthesis of Eumelanin–Silver–SiO_2_ Nanoparticles

All samples were produced following a previously described in situ protocol based on a sol–gel route.^25,27^ Different nominal amounts of DHICA and AgNO_3_ were used in order to investigate the effect of both parameters on the final properties of the nanoparticles. Employed quantities of DHICA and AgNO_3_ precursors, as well as molar ratio and the corresponding sample acronyms, are reported in Table 1. In a typical synthesis, an APTS-DHICA hybrid precursor was first synthesized coupling DHICA carboxyl (−COOH) groups to amino groups of APTS molecules through EDC/NHS chemistry. To this purpose, EDC (4.10 mg), NHS (28.5 mg), and APTS (5.00 μL) were added to a DHICA (4.13 mg) deaerated solution in ethanol (43.2 mL) and water (9.00 mL). The reaction was allowed to proceed under stirring for 15 min at 4 °C in an ice bath (solution 1). Then, solution 1 was removed from ice bath, and a proper amount of DHICA (4.13, 16.52, and 37.18 mg depending on the sample to be produced) was further added, in order to reach the nominal amount indicated in Table 1. Synthesis of hybrid nanoparticles was initiated at room temperature using a modified Stöber method. In summary, a mixture of TEOS (1.86 mL) in ethanol (23.2 mL) was added to solution 1, followed by the addition of the AgNO_3_ ethanol solution obtained by dissolving appropriate amounts of AgNO_3_ (1.46, 7.28, 36.40, and 54.60 mg, depending on the sample to be produced) and a mixture of ammonia in ethanol. After 18 h, obtained nanoparticles (NPs) were recovered by centrifugation and repeatedly washed with water. Produced nanoparticles will be named in the following as DHICAx-Agy@Sil NPs. More specifically, *x* is the ratio between the moles of DHICA used in the preparation of each hybrid sample and the moles of DHICA used in the synthesis of the first nanostructures presented by the authors in a previous paper, chosen as reference.^27^ Similarly, the meaning of *y* is the same except for Ag.

**Table 1:** Nominal Amount of DHICA and Silver in DHICA-Ag@Sil Nanoparticles

Systems	DHICA (mol·10^–5^)	Ag^+^ (mol·10^–5^)	DHICA/AgNO_3_ (mol/mol)	*x* (mol/mol)	*y* (mol/mol)
DHICA-Ag0.2@Sil	2.14	0.43	1/0.2	1	0.2
DHICA-Ag@Sil	2.14	2.14	1/1	1	1
DHICA-Ag5@Sil	2.14	10.7	1/5	1	5
DHICA-Ag7.5@Sil	2.14	16.1	1/7.5	1	7.5
DHICA2.5-Ag5@Sil	5.35	10.7	1/2	2.5	5
DHICA5-Ag5@Sil	10.7	10.7	1/1	5	5

### 2.3 Transmission Electron Microscopy (TEM)

For TEM analysis, about 10 μL of a solution containing nanoparticles was spread onto a copper grid (200 mesh with carbon membrane). TEM images were obtained using a TECNAI 20 G2, FEI Company with a camera Eagle 2HS. The images were acquired at 200 kV; camera exposure time = 1 s; size = 2048 × 2048.

### 2.4 Photoacoustic Experimental Setups

The multimodality imaging platform Vevo LAZR X (FUJIFILM VisualSonics Inc.) was used for the tests. The PA spectral analysis was performed from 680 to 970 nm and under prolonged laser illumination in test-object and ex vivo phantoms to study the photostability (PHSt) during time. The PA platform provided coregistered ultrasonographic and photoacoustic images, combining echographic probes with specific supports for the optical laser fiber to bring the laser light stimulation on the sample by two optical spots on the sides of the echographic probe. The PA system generated 6–8 ns laser pulses at a repetition rate of 20 Hz, and the laser was a Nd:YAG with an optical parametric oscillator (OPO), providing the tuning of the light wavelength. To check the performance of DHICA_*x*_-Ag_*y*_@Sil NPs, different dilutions were prepared, then loaded into polyethylene tubes (i.d. 560 μm, o.d. 990 μm). The polyethylene tubes minimize the optical and acoustic absorption thanks to their physical characteristics like optical transparency for light and useful acoustic impedance for ultrasounds. The tubes were placed complanarly in the same plane of PA acquisition to maintain the same geometrical configuration in terms of laser focusing inside a polypropylene box. This box was filled with water in order to optimize the acoustic coupling with the PA probe. Moreover, this configuration through water coupling allows one to manage and avoid the potential creation of air bubbles that could create ultrasound artifacts, capable of invalidating the measurements. Once the box was filled, the PA probe was immersed and placed at around 7 mm from the tubes, the distance for which the light focusing was optimized. The PA multispectral behavior and photostability were then evaluated. For the spectral analysis, each sample was irradiated in an optical window acquiring the PA signal intensity at each wavelength from 680 up to 970 nm with a 5 nm step, before and after the photostability tests. The photostability was assessed by maintaining the PA probe in the same position and keeping the samples under prolonged laser illumination at the fixed wavelength (705 nm) for more than 60 s, meaning over 300 laser light pulses of around 55 mJ for each one. All of the different dilution samples were analyzed following the same order: PA spectral analysis, PA photostability, and then again PA spectral analysis to verify if the prolonged laser illumination at a fixed wavelength changed the spectral properties. Indeed, for some kinds of nanoarchitectures, the pulsed light could change the optical response causing reshaping phenomena due to the released laser power (i.e., gold nanorods).

### 2.5 Electron paramagnetic resonance (EPR) spectroscopy

EPR spectroscopy experiments were carried out by means of an X-band (9 GHz) Bruker Elexys E-500 spectrometer (Bruker, Rheinstetten, Germany), equipped with a superhigh sensitivity probe head. Solid samples were transferred to flame-sealed glass capillaries, which, in turn, were coaxially inserted in a standard 4 mm quartz sample tube. Measurements were performed at room temperature. The instrumental settings were as follows: sweep width, 100 G; resolution, 1024 points; modulation frequency, 100 kHz; and modulation amplitude, 1.0 G. The amplitude of the field modulation was preventively checked to be low enough to avoid detectable signal over modulation. Preliminarily, EPR spectra were measured with a microwave power of ≃0.6 mW to avoid microwave saturation of the resonance absorption curve. Several scans, typically 32, were accumulated to improve the signal-to-noise ratio. Successively, for power saturation experiments, the microwave power was gradually incremented from 0.02 to 160 mW. The g-factor value was evaluated by means of an internal standard (Mg/MnO) which was inserted in the quartz sample tube coaxially with the capillary containing the samples. Free radical concentration in the sample was estimated by using a specific amount of the MnO sample as the reference. The area under the EPR absorption curves was estimated by the double integration of their first derivatives.

## 3 Results

To understand the differential contribution of silver and melanin, we standardized a few synthesis protocols leading to a different weight ratio of these components in the final nanoparticles as reported in Table 1. First, the amount of DHICA was kept constant at the value employed in a previous study,^27^ and samples at different Ag content were synthesized and indicated by the acronym DHICAx-Agy@Sil. All DHICAx-Agy@Sil NPs were characterized to quantify the localized light absorption and PA performances; then, a further set of samples was prepared with a nominal Ag content fixed at the value of the sample with the best PA response (DHICA-Ag5@Sil) and changing the DHICA amount in the synthesis batch, as detailed further down in Table 1.

Although the DHICA-Ag7.5@Sil sample showed the highest PA performance (as detailed below), this was not reproducible because of poor aggregation stability. Thus, this composition was no longer considered as relevant to optimize PA performance of DHICAx-Agy@Sil systems.

### 3.1 PA Imaging of Ag–Melanin Nanoparticles

**Figure 1:**
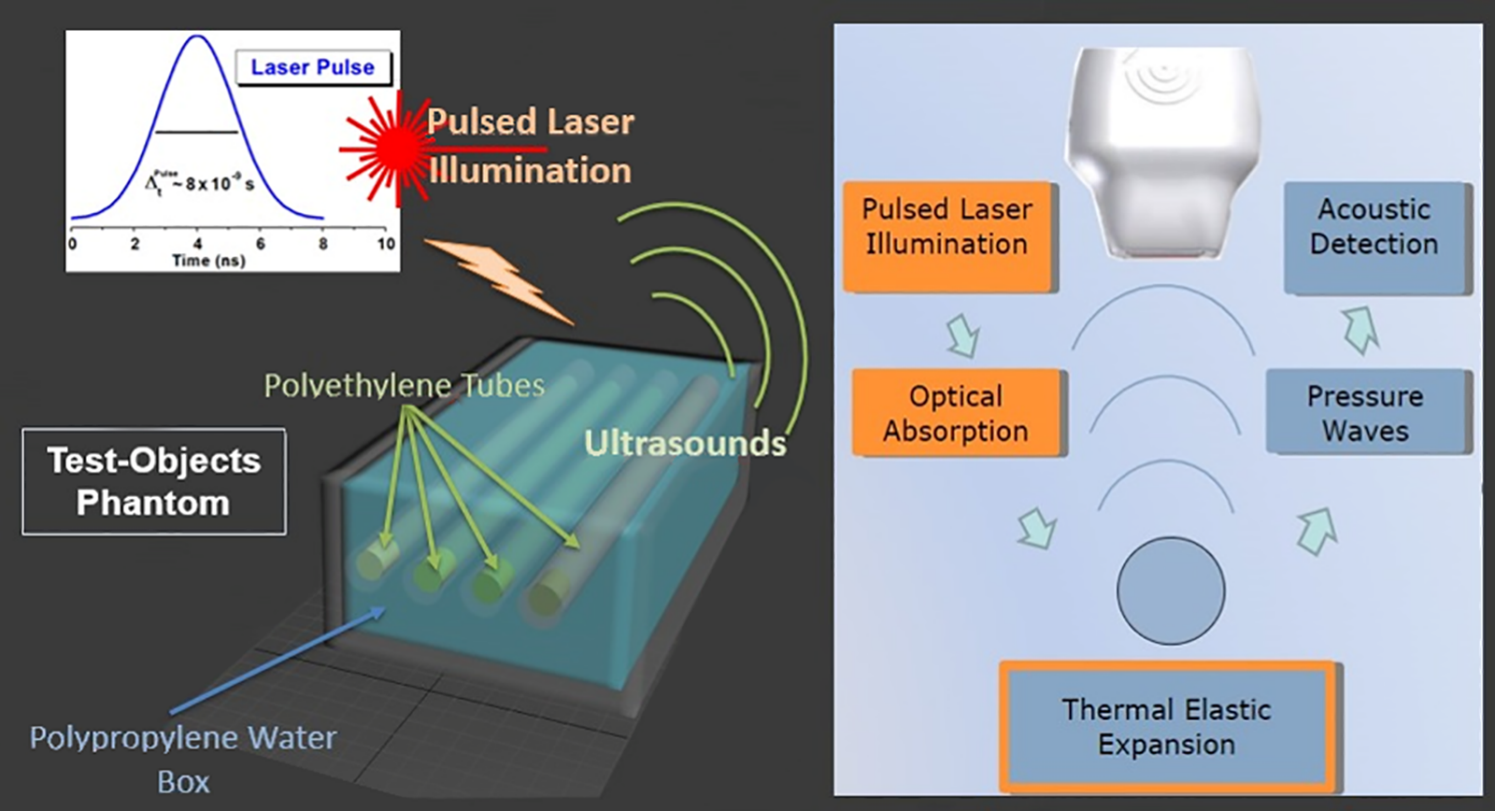
On the left side, the 3d rendering of the test-object phantom projected for the photoacoustic analysis; on the right side, the sketch of the cycle of the stimulation of the photoacoustic effect.

The photoacoustic performances of DHICAx-Agy@Sil NPs were measured using a custom setup^27,30^ (sketch in Figure 1) to study the correlation between composition and the localized effect under light illumination.

The PA results are shown in Figure S1, reporting the spectral PA response of DHICAx-Agy@Sil NPs at different silver amounts (0.2, 1, 5, and 7.5) (Figure S1A) and the photostability overtime under prolonged laser stimulation at 705 nm wavelength (Figure S1B). The samples were excited for over 80 s (over 500 laser pulses). DHICAx-Agy@Sil NPs with different Ag content underlined stability over all the time of stimulation with a percentage variation coefficient between 1.3 and 9.9 (Table S1), and the related signal-to-noise ratio (SNR) from 11 up to 76 (Table S1), highlighting their potential use as medical contrast agents.

In the same way, the PA results are inherent in the spectral PA responses of DHICAx-Agy@Sil nanoparticles with a constant amount of silver, and DHICA variable amounts are reported in Figure S1A′ and B′. Plots in Figure S1A′ and B′ show the PA spectral responses with pre- and post-prolonged laser stimulation. Their comparison, as explained before, showed a decrease of the PA signal intensity 0.35 a.u.

The evaluation of the photostability of DHICAx-Agy@Sil NPs with a constant amount of silver stimulated by prolonged laser illumination at 705 nm showed that DHICAx-Agy@Sil nanoparticles underlined stability over all the time of stimulation with a percentage variation coefficient between 0.59 and 1.87 (Table S1), and the related signal-to-noise ratio (SNR) was 170 (Table S2).

**Figure 2:**
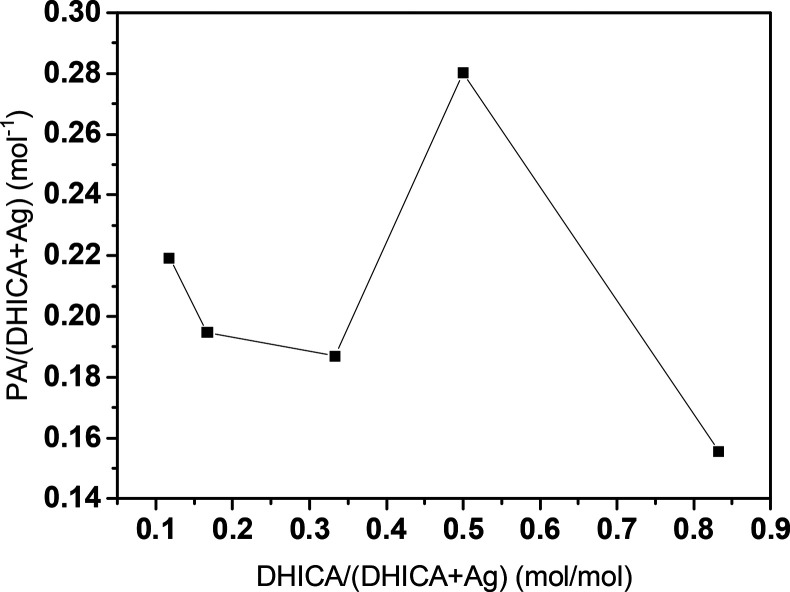
Normalized PA signal (error < 5%) against the overall mole amount of the active phase (silver + DHICA) vs DHICA molar ratio in the active phase.

According to the nominal amount of Ag and DHICA (see Table 1), the PA signal normalized to the total molar amount of the active components (i.e., Ag and DHICA) vs DHICA molar fraction in the PA chromophores; i.e., DHICA/(DHICA+Ag) of each sample was plotted (Figure 2).

The plot shows a continuous trend with a peak for the systems containing equimolar content in DHICA and silver (DHICA-Ag@Sil, DHICA5-Ag5@Sil), suggesting a synergic effect between silver and DHICA in those compositions, concurring with the enhancement of the PA signal.

In order to unveil the role played by each component in generating the PA signal, a detailed physical–chemical investigation was carried out on the prepared samples and integrated with a physical model which could account for obtained results from both physicochemical characterization and PA performance.

Electron paramagnetic resonance (EPR) analysis (Figures S2 and S3) indicated in all cases a typical melanin-type signal with a g-factor value similar to that of the previously reported silver-free nanoparticles (*g* = 2.0035)^25^ and exhibiting a modest, yet nonsignificant, decrease with an increasing Ag/DHICA ratio (Table S3), which could be ascribable to either a higher degree of melanin polymerization or a prevalence of carbon-centered vs oxygen-centered radicals. Similar changes in the *g*-factor were observed with changing the DHICA concentration while keeping Ag fixed (Table S4). The same values of ΔB for both DHICA-Ag@Sil and DHICA5-Ag5@Sil samples (Tables S3 and S4) could suggest a very similar supramolecular organization of the melanin pigment driven by a molar ratio equal to 1:1 mol, confirming the best chemical condition realizing the nanosystems with high performances.

### 3.2 TEM Analysis

**Figure 3:**
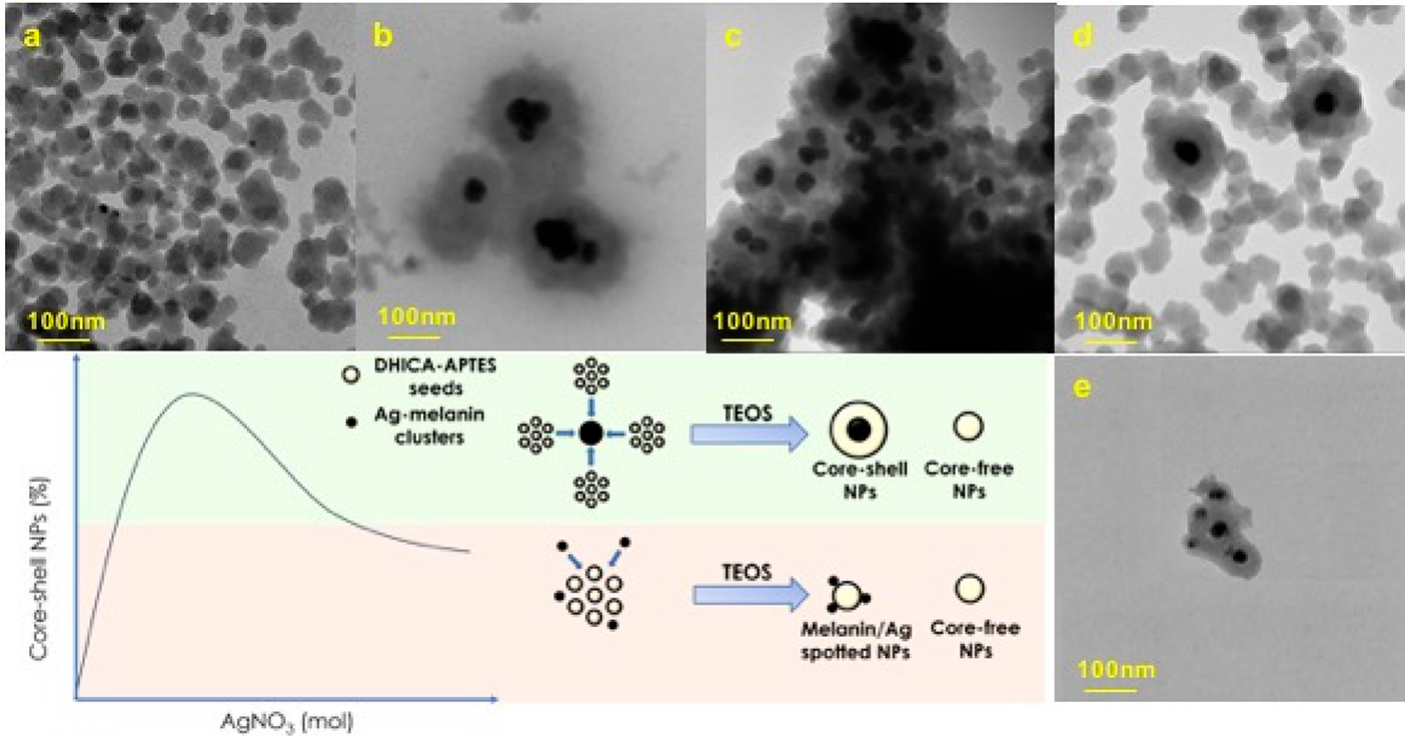
TEM micrographs of DHICA-Ag0.2@Sil (a), DHICA-Ag@Sil (b), DHICA-Ag5@Sil (c), DHICA2.5-Ag5@Sil (d), and DHICA5-Ag5@Sil (e) NPs and proposed formation mechanism of hybrid nanosystems.

The particle structure was examined by transmission electron microscopy (TEM). Images of all synthesized samples are reported in Figure 3 and Figure S4 (at lower magnification).

TEM images evidence that silver and DHICA molar ratios markedly influence both size and structure of hybrid systems. Increasing the Ag^+^/DHICA ratio at constant DHICA concentration resulted in the assembly of well-defined pseudo-core–shell structures in proportions and size depending on the silver ion load in the synthesis batch. Table 2 reports both the particle and core average sizes derived from TEM analysis, the Ag^+^/DHICA molar ratio, and the estimated percentage of core–shell particles in the total population. The average particle size follows a nonmonotonic trend with silver content in the samples; the largest value (140–150 nm) was obtained for the Ag^+^/DHICA equimolar amount which showed the largest normalized PA signal (DHICA5-Ag5@Sil, DHICA-Ag@Sil NPs). Exceeding the 5/1 ratio resulted in a decrease in structural integrity with massive aggregation because of the insufficient melanin-based component putatively controlling the core–shell architecture. Obtained sizes are in fair accordance with those obtained by DLS measurements (Figure S5). Larger values assessed in the latter case might be ascribed to large size distribution, hydration phenomena in aqueous solution due to the hydrophilic silica shell, and even to formation of reversible aggregates in suspension.^31−33^

**Table 2:** Particle and Core Average Sizes of NPs Derived from TEM Analysis, Silver/DHICA Molar Ratio, and Estimated Percentage of Core–Shell Particles in the Total Population of All Samples

Systems	NP size (nm)	Core size (nm)	Ag^+^/DHICA (mol/mol)	% core shell type NP
DHICA-Ag0.2@Sil	40	NE	0.2	–
DHICA-Ag@Sil	150	42	1	19
DHICA-Ag5@Sil	80	32	5	13
DHICA2.5-Ag5@Sil	140	30	2	5
DHICA5-Ag5@Sil	130	33	1	6

Moreover, TEM images of each sample show the presence of a large population of core-free nanostructures, about 30 nm in size, which is not influenced by melanin and silver content. Indeed, these nanostructures could play a key role in building the outer layer of core–shell nanostructures, since at a deeper insight, they show a petal morphology (Figure 3b, d), suggesting that it was produced by aggregation of primary particles. Fixing the Ag^+^ concentration as in the DHICA-Ag5@Sil sample, the DHICA/Ag^+^ ratio was then varied, and the impact on the formation of core–shell structures was determined. TEM micrographs indicated an increase in the density of core–shell structures within the NP population (Figure 3a–c). At a closer look, the dark core appears as it is made by a few darker dots embedded into a lighter region (Figure 3a, d), suggesting that it could be composed of a Ag and melanin intimate mixture, obtained by virtue of the peculiar formation mechanism wherein both components play a key role in their reciprocal development, as described below.

By matching the morphological features of the obtained nanostructures with PA performance, it can be argued that this is related to the fraction of core–shell nanoparticles as well as their size. Actually, the samples with the largest NP size produced the highest PA signal. This result is in accordance with previous studies, which reported that aggregation of melanin-based nanoparticles into larger clusters resulted in PA signal amplification.^34^ Morphological and structural features of the prepared samples suggest that their formation could be based on heterogeneous nucleation processes. Notably, on the basis of TEM evidence and the synthesis procedure, a likely formation mechanism of DHICAx-Agy@Sil nanostructures can be sketched (Figure 3, formation mechanism panel). The first synthesis step involves production of the APTS-DHICA hybrid precursor through EDC chemistry. Since the coupling reaction is carried out in water, APTS concurrently undergoes hydrolysis and condensation, thus producing hybrid seeds for the development of both organic and inorganic components, which occurs by growth and aggregation processes in the following stages. Upon addition of further amounts of DHICA and AgNO_3_ to the synthesis batch, the reaction of DHICA with Ag^+^ ions results in the oxidative conversion of indole to melanin-type oligomers with the concomitant reduction of Ag^+^ to metallic Ag nanoclusters;^27^ the pattern reported in Figure S6, representative of all samples (spectra not shown), shows a wide diffraction peak at about 22° (2θ) corresponding to the amorphous silica and four diffraction peaks attributed to face-centered-cubic (fcc) metallic Ag,^35^ thus proving silver reduction and a supporting redox reaction between Ag+ ions and DHICA, accounting for melanin formation. Since Ag^+^ ions trigger melanin precipitation, an intimate mixture between the two components is envisaged, with melanin oligomers surrounding and immobilizing the growing metallic dots. According to a widely recognized melanin formation mechanism, oligomers build up a more complex structure by aggregation,^36^ which is expected to preferentially occur on hybrid seeds produced in the first step. On the other hand, upon TEOS addition, its hydrolysis and condensation reactions produce a large population of primary particles which grow by aggregation. Since TEOS is the most abundant precursor in the reaction environment, this process could account for the great number of core-free nanostructures in all prepared samples. Given that nanoparticle growth is bound to occur by aggregation of small nanostructures to the larger ones,^37^ nanoparticle morphology and structure result from the competition of the described processes. In particular, at low silver content, the formation of the melanin/metal-integrated component is slow, and hybrid seeds are mainly involved in the formation of the silica phase, which occurs fast because of TEOS abundance. Thus, melanin/Ag oligomers aggregate onto silica domains leading to isolated surface dark spots (Figure 3, formation mechanism panel). On the other hand, at relevant silver and DHICA content, DHICA oxidative polymerization occurs faster than silica formation because of rapid red-ox kinetics, producing a relevant fraction of hybrid melanin–silver clusters which grow by aggregation onto hybrid seeds. According to the small-to-large aggregation mechanism,^37^ silica primary particles preferentially grow by aggregation onto melanin–silver nanostructures resulting into a self-assembled core–shell architecture. The petal-like morphology of the outer layer (Figure 3b,d) confirms the hypothesis that it is made by the aggregation of primary particles. In addition, from the analysis of TEM images, it can be inferred that the silica component plays a role by isolating the interacting Ag^+^–DHICA components, limiting their further stacking and controlling hybrid particle growth.

Based on the results of the TEM images, it can be argued that an important requisite for both efficient absorbance and PA signal emission is the presence of intact and well separated core–shell structures.

In order to support this hypothesis and account for the interaction of melanin and silver in the enhancement of the PA signal, a model based on the Mie scattering approach was developed to calculate the extinction cross-section of prepared NPs.

### 3.3 Signal Enhancement Phenomena Provided from Core–Shell DHICA-Ag Nanoarchitectures in DHICAx-Agy@Sil NP

Now, we analyze the origin of the PA, calculate the light absorption with numerical simulations, and compare it with the measured PA obtained in the previous sections. The enhancement of the PA signal, as we see, is due to the tail of a red-shifted plasmonic resonance due to melanin’s presence; such resonances provide a high extinction cross-section of the particles. For small metal nanoparticles, absorption and extinction cross-section are almost equal;^27,28^ in addition, the PA signal is proportional to the absorption cross-section, as shown by Rapenko et al.^38^ In the limit of small concentrations, the PA signal is given by PA = *kσ*, where σ is the absorption cross-section, and *k* is a constant that depends on the concentration, incident power, and particle mass.^38^ This means that an enhancement in absorption corresponds to enhancements in the PA signal.

Plasmon resonances are oscillations of the electron densities in the metal nanoparticles driven by an incident laser (light) that have a resonant behavior for particular frequencies.^27^ The architecture of the DHICAx-Agy@Sil NPs results in a complex aggregate where a Ag core is covered by melanin, as can be seen in the TEM images of Figure 3. In order to gain insight on the plasmonic response of such structures, we approximate them as spherical core–shell structures, where a Ag core is covered by a melanin layer.

**Figure 4:**
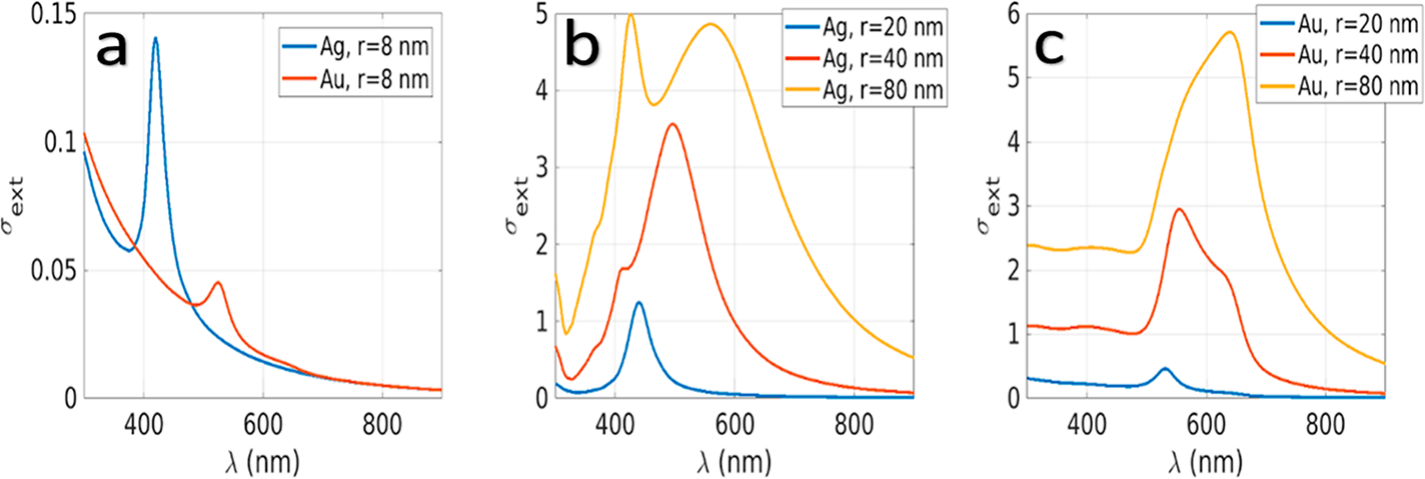
Extinction cross-section of silver and gold nanoparticles embedded in melanin with different dimensions: *r* = 8 nm (a); *r* = 20–40–80 nm Ag (b); *r* = 20–40–80 nm Au(c). The nanoparticles are at the center of a melanin particle of 140 nm that is in water.

Indeed, as confirmed by computer simulations based on the Mie scattering approach^28^ (Figure 4), the obtained PA signal is explained by a hybrid structure wherein melanin incorporates a silver cluster of silver nanospheres of the order of tenth nanometers. This outcome is also confirmed by the nanoparticle structure obtained from TEM images, supporting the perception that the inner dark core of DHICAx-Agy@Sil nanoparticles is a hybrid cluster of smaller nanodomains (Figure 3). The melanin environment is crucial to get the surface plasmonic resonance by laser stimulation closer to the near-infrared wavelengths where our laser operates. Indeed, melanin changes the refractive index around the silver nanoparticles. This change leads to a spectral red-shift of the plasmon resonance.^29^

**Figure 5:**
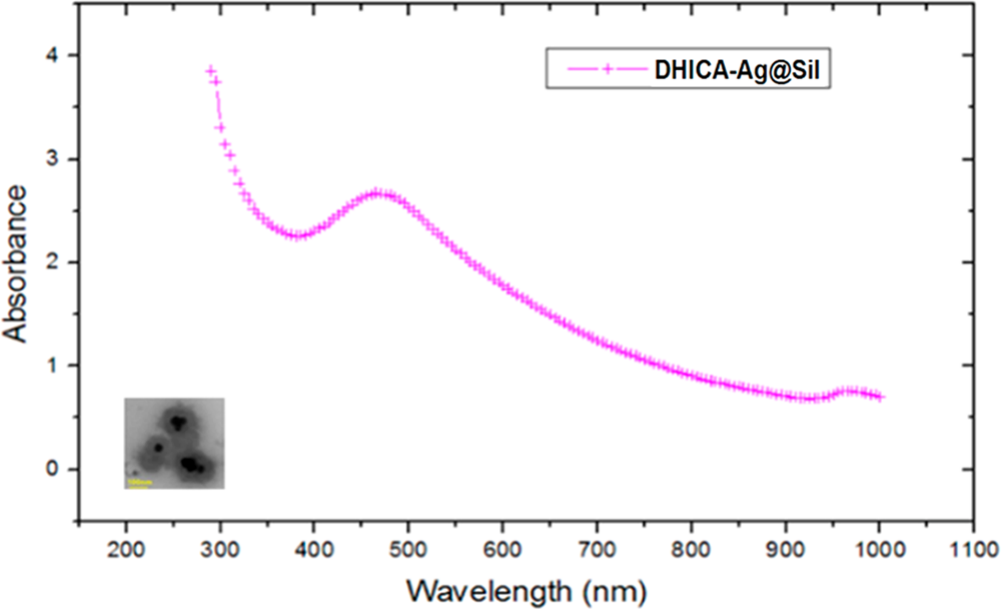
Spectrophotometer analysis of nanoparticles in the whole ultraviolet range from 200 to 1000 nm.

In Figure 4a (blue curve), we calculate the plasmonic resonance of a typical structure we have in our experiment (see TEM images in Figure 3). We can observe a clear peak around 430 nm (without melanin it will be around 350 nm) that will be the ideal frequency where we should have our laser pump. Anyway, the tail of such resonance is still enough to obtain a good PA signal, as we can observe in the multispectral trend plots in Figure S1A and A′, and the related photostability calculated at 705 nm (Figure S1B and B′). Furthermore, we can appreciate a good agreement between the simulated cross section in Figure 4a (blue curve) and the measured cross section shown in Figure 5.

Plasmonic resonances provide a strong electromagnetic field enhancement.^26,30^ The red-shift, induced by the melanin environment, enables a stronger response at 700 nm than that obtained by neat silver nanocluster with comparable size. This allows good excitation of the surface plasmon resonance in silver nanospheres even with laser light of around 700 nm. Another aspect to consider is efficiency in terms of energy released through heating. The intensity of the photoacoustic signal (PA) is related to the thermoelastic effects induced in the surrounding environment, which are related to the ability of the nanoparticle to transform the absorbed light energy into heat.^31,32^ The presence of agglomerates (particles with small gaps) allow further electromagnetic enhancement.^33^ In other words, it is well-known that small gaps between nanoparticles make possible the excitation of electromagnetic hotspots which are another mechanism favorable to the photoacoustic effect.^29^

In order to estimate the extent to which we can improve the PA contrast, we studied the effect of the nanoparticle size and material to enhance the photoelastic effect. In that direction, we used the Mie theory^34^ to analyze the plasmonic resonances for silver and gold when covered with a melanin shell with different dimensions. Figure 4 shows the extinction cross-section of particles with an 8 nm radius contained in a 140 nm nanoparticle sphere, as we investigated experimentally in this work, as well as the extinction cross sections of Ag and Au particles with bigger dimensions. We can appreciate that Ag presents a stronger plasmonic response than gold near 400 nm, in agreement with the experimental results of Figure 5, and for both materials, the extinction decay is at a longer wavelength as expected.

Such plasmonic resonances are enough good to enhance the photoelastic effect when we use a near-infrared laser but could still be hugely improved as we see in Figure 4b and c. Bigger nanoparticle dimensions present a stronger and red-shifted plasmonic resonance due to the larger effective plasmon wavelength and the radiative effects.^27^ In particular, bigger gold nanoparticles have a more red-shifted resonance, allowing better performances with a laser in the visible and near-infrared. Also, it is worth noting that in Figure 4b two peaks (yellow curve) are clearly appearing, and such peaks correspond with the dipolar and quadripolar plasmonic resonances.

## 4 Conclusions

Extensive physicochemical characterization and a physical model provided for an integrated approach to the origin of the potent PA response of ternary hybrid DHICAx-Agy@Sil NPs reported herein. Experimental results and theoretical data supported the operation of a delicate interplay of physical and chemical factors both within the Ag–melanin core–silica shell architecture and at the core metal–polymer interface. In particular, the marked increase in the PA signal with an increasing Ag/DHICA molar ratio followed by a drop beyond a critical threshold, which was accompanied by an increase in hybrid NP aggregation and partial loss of structural regularity, indicated that a well-defined and little aggregated core–shell architecture is an important requirement for an optimal PA response. Furthermore, increasing both DHICA and Ag^+^ was a valuable means of simultaneously enhancing melanin formation and Ag precipitation without affecting the core–shell architecture. The proposed formation mechanism accounts for the key role of each component in causing a self-assembly process into a core–shell architecture with an enhanced PA performance.

The spontaneous self-assembly of this latter architecture is a consequence of the competition between the formation and growth of primary particles in the presence of hybrid nuclei. Relevant DHICA and Ag content, particularly at equal molar ratios, speed up the primary redox reaction causing coprecipitation of metallic Ag and the melanin NPs. It is possible that just-produced primary Ag–melanin solid particles assemble into larger structures leading the deposition of SiO_2_ primary particles, which limit further particle growth. In this process, the silica matrix plays a fundamental role in keeping the growing Ag–melanin cores separated and perfectly adapted within the relatively large NP shell. In this setting, excess Ag^+^ over DHICA apparently disrupts the critical structural organization by promoting the excessive proliferation of growing metal–polymer nuclei overcoming the effect of silica which would no longer be able to prevent aggregation. In light of the redox nature of the Ag^+^–DHICA interaction, leading to the fast deposition of Ag–melanin particles, it is also concluded that DHICA polymerization to melanin does not occur at sites with depletion of Ag^+^ ions. This implies that relatively little or no Ag-free melanin is expected to be present in the DHICAx-Agy@Sil NPs and that, hence, the TEM visible cores are neither pure Ag nor pure melanin but intimate organic–inorganic hybrid substructures. This combination seems to account for more pronounced optical properties than each isolated component. In the present case, the hybrid system would especially benefit from the metal component partly reinforcing the inherently limited visible/NIR extinction coefficients of DHICA oligomers/melanin diluted in the silica matrix.^27^ It is reasonable, in this connection, that the intimate melanin–Ag interaction, implying a large and efficient bulk interface, provides synergistic effects involving the melanin component, which serves as a thermal insulator, efficiently transmitting the generated heat to the silver component. Consistent with the known importance of thermal confinement effects, it can be further suggested that the core–shell architecture allows efficient confinement of the active hybrid sonophore, ensuring increased temperature amplitudes and augmented photoacoustic contrast. The positive effect due to the plasmonic resonance has been elucidated, and further improvements using gold with bigger dimensions have been suggested through theoretical model.

In conclusion, we have shown that the DHICAx-Agy@Sil hybrid is a versatile platform that can be purposefully used to implement innovative, completely biocompatible, and finely tunable nanosystems via the rational inclusion of active ingredients and additives modulating or boosting the PA response via proper manipulation of the melanin–Ag interface. Particularly, the peculiar combination of silica, melanin, and silver makes the last an unprecedented highly performant contrast agent for the PA signal. Work is underway to probe the potential of the system for photothermal and theranostic applications.

## Supporting Information Available

PA characterization in PE tubes of DHICA_Ag nanoparticles with different Ag amounts: PA spectral trend of Ag_melanin nanoparticles acquired from the PE tubes in long axes geometry and photostability under prolonged laser stimulation at 705 nm fixed wavelength over time (Figure S1). DHICA-Ag@Sil nanoparticles in vitro test: PA mean values obtained during photostability investigations by illuminating at 705 nm, standard deviation, percentage variation coefficient, and signal-to-noise ratio (Table S1). Ag–melanin nanoparticles in vitro test: PA mean values obtained during photostability investigations by illuminating at 705 nm, standard deviation , percentage variation coefficient, and signal-to-noise ratio (Table S2). EPR spectra and power saturation curves of synthesized samples (Figure S2). Spectral parameter for synthesized samples obtained for EPR spectra reported in Figure S2 (Table S3). EPR spectra and power saturation curves of synthesized samples (Figure S3). Spectral parameter for synthesized samples obtained for EPR spectra reported in Figure S3 (Table S4). TEM micrographs of DHICA-Ag0.2@Sil, DHICA-Ag@Sil, DHICA-Ag5@Sil, DHICA2.5-Ag5@Sil, DHICA5-Ag5@Sil NPs (Figure S4). DLS data of synthesized samples (Figure S5). XRD Pattern representative of all DHICAx-Agy@Sil samples (Figure S6). (PDF)
