# Supplementary material for: Enhanced Photoacoustic Response by Synergistic Ag–Melanin Interplay at the Core of Ternary Biocompatible Hybrid Silica-Based Nanoparticles
Source: ACS Appl Mater Interfaces. 2023 Sep 29;15(40):46756–64. doi: 10.1021/acsami.3c13523 (PMC10571004; doi:10.1021/acsami.3c13523)
Supplement: Supplementary file 1 — am3c13523_si_001.pdf [file am3c13523_si_001.pdf]

Supplementary information

Enhanced Photoacoustic Response by Synergistic Ag-Melanin Interplay at the Core of Ternary Biocompatible Hybrid Silica-Based Nanoparticles

Brigida Silvestri[1‡], Paolo Armanetti[2‡], Giulio Pota[3‡], Giuseppe Vitiello[3,4], Alessandro Pezzella[5,6,7], Luca Menichetti[2*], Vincenzo Giannini[8,9*], Giuseppina Luciani[3*]

[1] Department of Civil, Architectural and Environmental Engineering, University of Naples Federico II, Via Claudio 21, 80125 Fuorigrotta, Naples, Italy

[2] Institute of Clinical Physiology, National Research Council, Via Giuseppe Moruzzi 1, 56124 Pisa, Italy

[3] Department of Chemical, Materials and Production Engineering, University of Naples “Federico II”, p.le V. Tecchio 80, 80125 Naples, Italy

[4] CSGI, Consorzio interuniversitario per lo sviluppo dei Sistemi a Grande Interfase, Sesto Fiorentino, via della Lastruccia 3, 50019 Firenze, Italy

[5] National Interuniversity Consortium of Materials Science and Technology (INSTM), Via G. Giusti 9, 50121 Florence, Italy

[6] Institute for Polymers, Composites and Biomaterials (IPCB), CNR, Via Campi Flegrei 34, I-80078 Pozzuoli (NA), Italy

[7] Department of Physics Ettore Pancini, University of Naples “Federico II” Via Cintia 4, I-80126 Naples Italy

[8] Instituto de Estructura de la Materia (IEM), Consejo Superior de Investigaciones Científicas (CSIC), Serrano 121, Madrid, 28006, Spain

[9] Technology Innovation Institute, Building B04C, P.O. Box, Abu Dhabi, 9639, United Arab Emirates

*Corresponding Authors: luciani@unina.it; luca.menichetti@cnr.it; v.giannini@csic.es

Contents

Photostability testing during pulsed irradiation 3

Figure S1: PA characterization in PE tubes of DHICA_Ag nanoparticles with different Ag amounts: (A, A') PA spectral trend of Ag_melanin nanoparticles acquired from the PE tubes in long axes geometry; (B ,B') Photostability under prolonged laser stimulation at 705 nm fixed wavelength over time. 3

Table S1: DHICA-Ag@Sil nanoparticles (%Ag) in vitro test: PA mean values obtained during photostability investigations by illuminating at 705 nm, standard deviation (Std.Dev), percentage variation coefficient (%CV), and signal to noise ratio (SNR). 3

Table S2: Ag-Melanin nanoparticles (%Ag) in vitro test: PA mean values obtained during photostability investigations by illuminating at 705 nm, standard deviation (Std.Dev), percentage variation coefficient (%CV), and signal to noise ratio (SNR). 4

***Electron paramagnetic resonance (EPR) spectroscopy*......4**

Figure S2. EPR spectra (left side) and power saturation curves (right side) of synthetized samples. 4

Table S3. Spectral parameter for synthetized samples obtained for EPR spectra reported in Figure S2. 4

......4

Figure S3. EPR spectra (left side) and power saturation curves (right side) of synthetized samples. 4

Table S4. Spectral parameter for synthetized samples obtained for EPR spectra reported in Figure S3. 4

Morphological Investigation 5

Figure S4. TEM micrographs of DHICA-Ag0.2@Sil (a), DHICA-Ag@Sil (b), DHICA-Ag5@Sil (c), DHICA2.5-Ag5@Sil (d), DHICA5-Ag5@Sil (e) NPs**.** 5

Dynamic light scattering (DLS) investigation 5

Figure S5. DLS data of synthesized samples. 5

XRD Analysis 6

Figure S6. XRD Pattern representative of all DHICA$_x$-Ag$_y$@Sil samples. 6

Photostability testing during pulsed irradiation

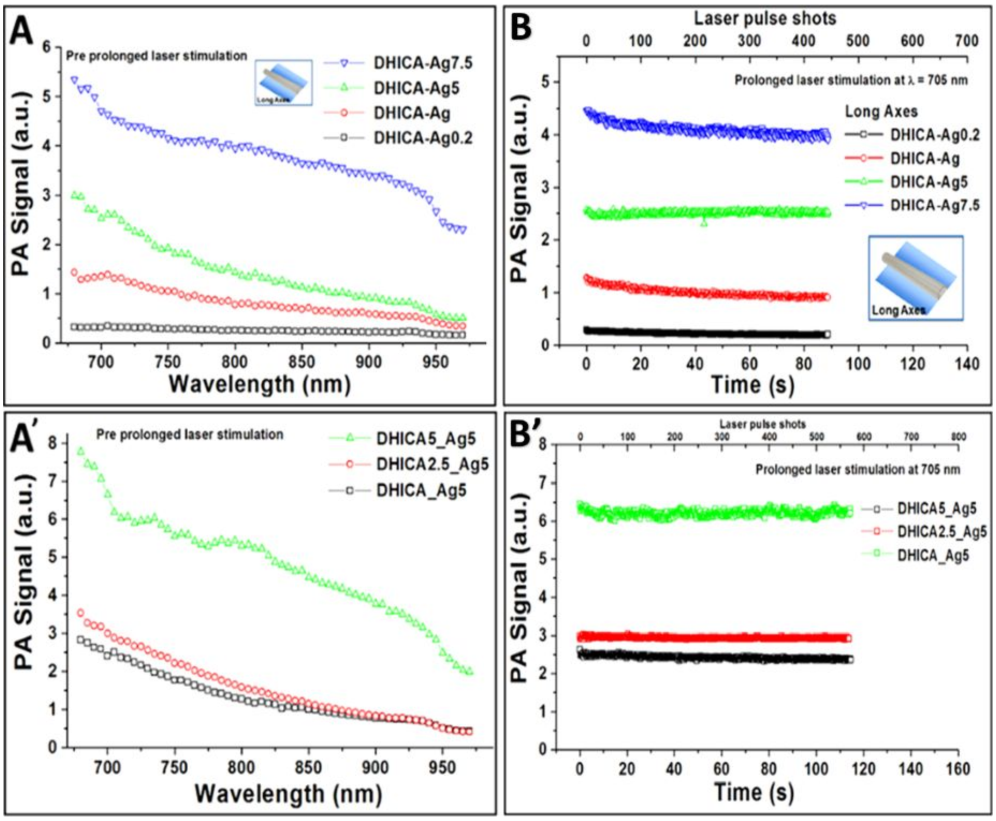

Figure S1: PA characterization in PE tubes of DHICA_Ag nanoparticles with different Ag amounts: (A, A') PA spectral trend of Ag_melanin nanoparticles acquired from the PE tubes in long axes geometry; (B ,B') Photostability under prolonged laser stimulation at 705 nm fixed wavelength over time.

Table S1: DHICA-Ag@Sil nanoparticles (%Ag) in vitro test: PA mean values obtained during photostability investigations by illuminating at 705 nm, standard deviation (Std.Dev), percentage variation coefficient (%CV), and signal to noise ratio (SNR).

DHICA-Ag@Sil (%Ag)	*PA Signal (a.u.)*	*Std.Dev.*	*%CV*	*SNR*
0.2	*0,22*	*0.02*	9,92	*11*
1	*1,00*	*0.08*	*8,37*	*12*
5	*2,51*	*0.03*	*1,32*	*76*
7.5	*4,10*	*0.011*	*2,85*	*39*

Table S2: Ag-Melanin nanoparticles (%Ag) in vitro test: PA mean values obtained during photostability investigations by illuminating at 705 nm, standard deviation (Std.Dev), percentage variation coefficient (%CV), and signal to noise ratio (SNR).

Dhica_Ag Nps (%Dhica)	***PA Signal (a.u.)***	***Std.Dev.***	***%CV***	***SNR***
2	*2,42*	*0,04*	*1,87*	*53*
5	*2,94*	*0,02*	*0,59*	*170*
10	*6,20*	*0,07*	*1,15*	*87*

Electron paramagnetic resonance (EPR) spectroscopy

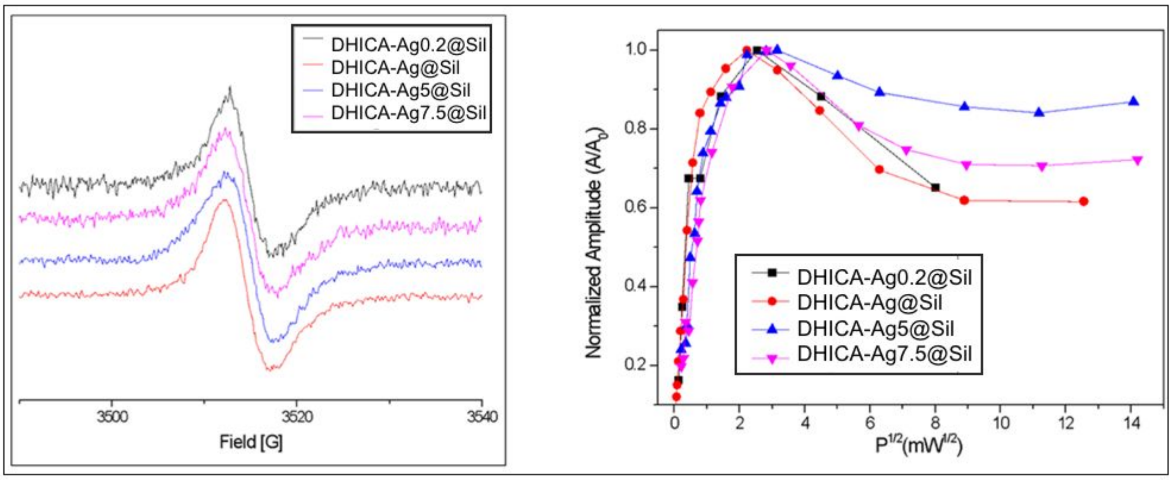

Figure S2. EPR spectra (left side) and power saturation curves (right side) of synthetized samples.

Table S3. Spectral parameter for synthetized samples obtained for EPR spectra reported in Figure S2.

**NPs**	**ΔB ± 0.1**	**g factor ± 0.0003**
SiO_2-DHICA-Ag7.5	5.7	2.0033
SiO_2-DHICA-Ag5	5.5	2.0034
SiO_2-DHICA-Ag	5.3	2.0036
SiO_2-DHICA-Ag0.2	5.5	2.0037

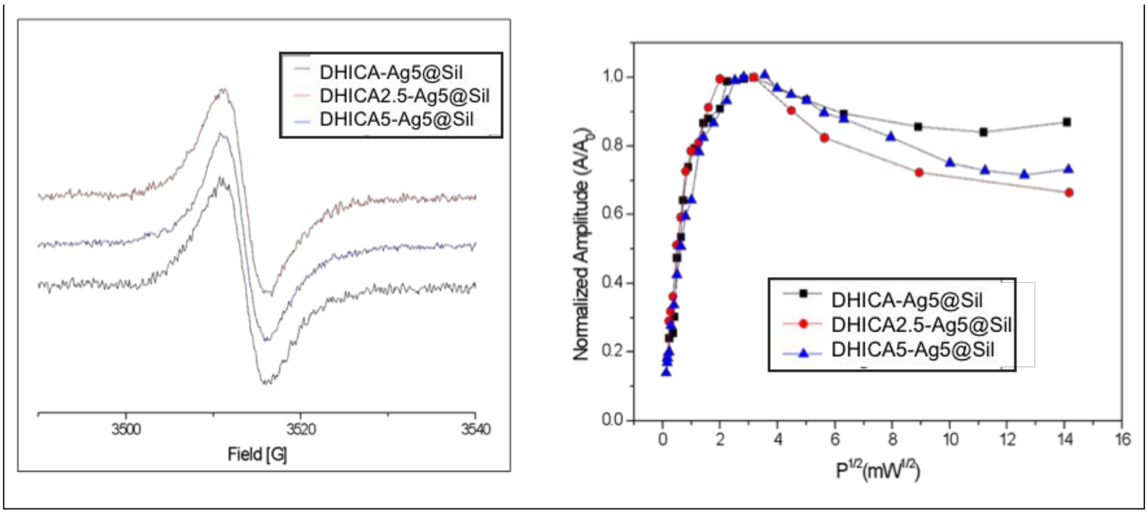

Figure S3. EPR spectra (left side) and power saturation curves (right side) of synthetized samples.

Table S4. Spectral parameter for synthetized samples obtained for EPR spectra reported in Figure S3.

**NPs**	**ΔB ± 0.1**	**g factor ± 0.0003**
SiO_2-DHICA-Ag5	5.4	2.0034
SiO_2-DHICA2.5-Ag5	5.1	2.0032
SiO_2-DHICA5-Ag5	5.3	2.0036

Morphological Investigation

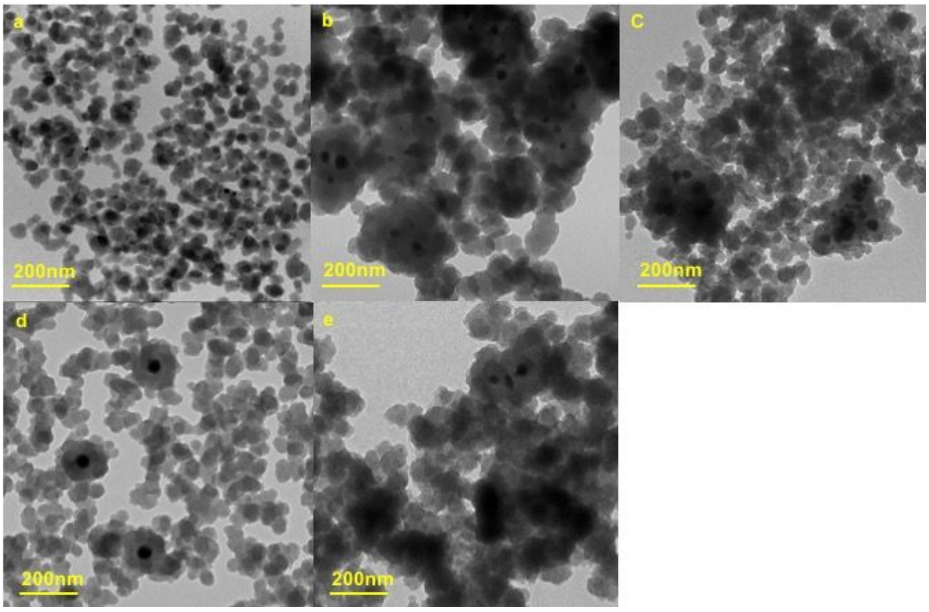

Figure S4. TEM micrographs of DHICA-Ag0.2@Sil (a), DHICA-Ag@Sil (b), DHICA-Ag5@Sil (c), DHICA2.5-Ag5@Sil (d), DHICA5-Ag5@Sil (e) NPs.

Dynamic light scattering (DLS) investigation

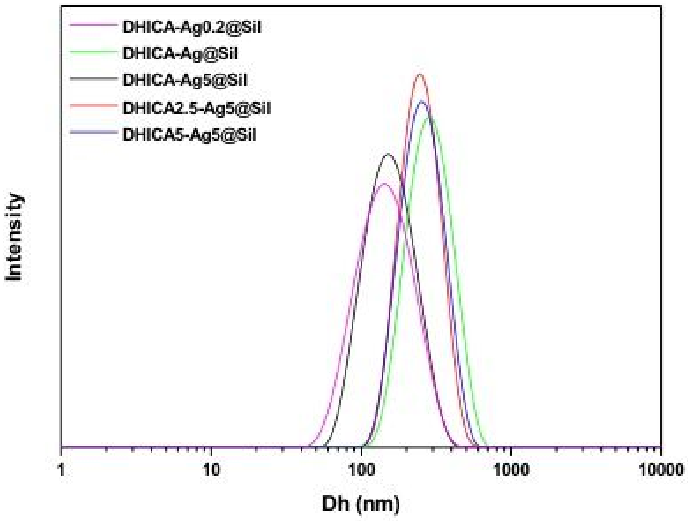

Figure S5. DLS data of synthesized samples.

XRD Analysis

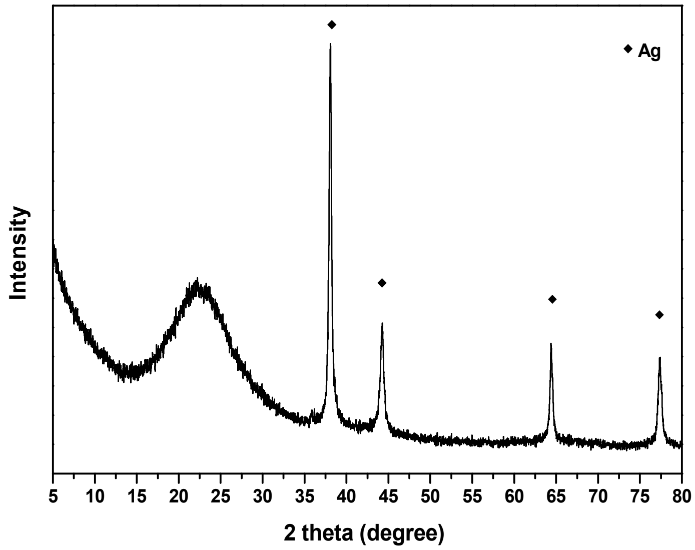

Figure S6. XRD Pattern representative of all DHICA$_x$-Ag$_y$@Sil samples.
